# Long-term performance trajectories in classic powerlifting: an exploratory analysis of 6,524 lifters with sustained competitive engagement

**DOI:** 10.1186/s13102-026-01775-w

**Published:** 2026-05-29

**Authors:** Alexander Renner, Daniel van den Hoek, Robert Csapo

**Affiliations:** 1 Centre for Sport Science and University Sports, Department of Sport and Human Movement Science, University of Vienna, Auf der Schmelz 6, Vienna, 1150 Austria; 2 School of Health, University of the Sunshine Coast, Sippy Downs, QLD Australia

**Keywords:** Classic powerlifting, Long-term performance, Career trajectories, Peak age, Competition frequency, Attempt success rate, Weight-class changes, Talent benchmarking

## Abstract

**Background:**

Previous powerlifting research has largely examined correlates of performance in single competitions or over short observation periods, leaving long-term patterns of athletic development among sustained competitors insufficiently described. The present investigation addresses this gap by examining performance progression, observed peak performance timing, and correlates of peak career success in classic powerlifting.

**Methods:**

This retrospective observational study analyzed IPF-affiliated classic powerlifters with at least 10 completed competitions. Population-level age-performance patterns were described using LOESS smoothing with a lifter-level cluster bootstrap. Between-group differences in annual performance progression were examined using multivariable ordinary least squares models with lifter-clustered standard errors. Associations with observed peak timing were examined using multivariable ordinary least squares models, complemented by discrete-time event-history robustness analyses, and correlates of peak career performance were examined using multivariable ordinary least squares models.

**Results:**

Within this selected cohort, annual gains generally declined with increasing years in sport. Peak performance occurred at approximately 27 years of age and observed individual peak performance was typically reached after 4–6 years of competition participation, around the 10th competition. Higher competition frequency was associated with earlier observed peak timing when indexed by year in sport, but with later observed peak timing when indexed by competition number and was not associated with greater peak performance. Age and IPF GL Points at the first competition were the strongest multivariable correlates of observed peak career performance, whereas attempt success rate, sex, and weight-class change patterns showed statistically significant but comparatively small effects.

**Conclusions:**

Early competitive performance and age at entry were strong multivariable correlates of observed peak career performance, while higher competition frequency appeared to relate primarily to the pacing and competitive exposure of development rather than to peak magnitude. These findings may inform early benchmarking and individualized long-term competition planning.

**Graphical abstract:**

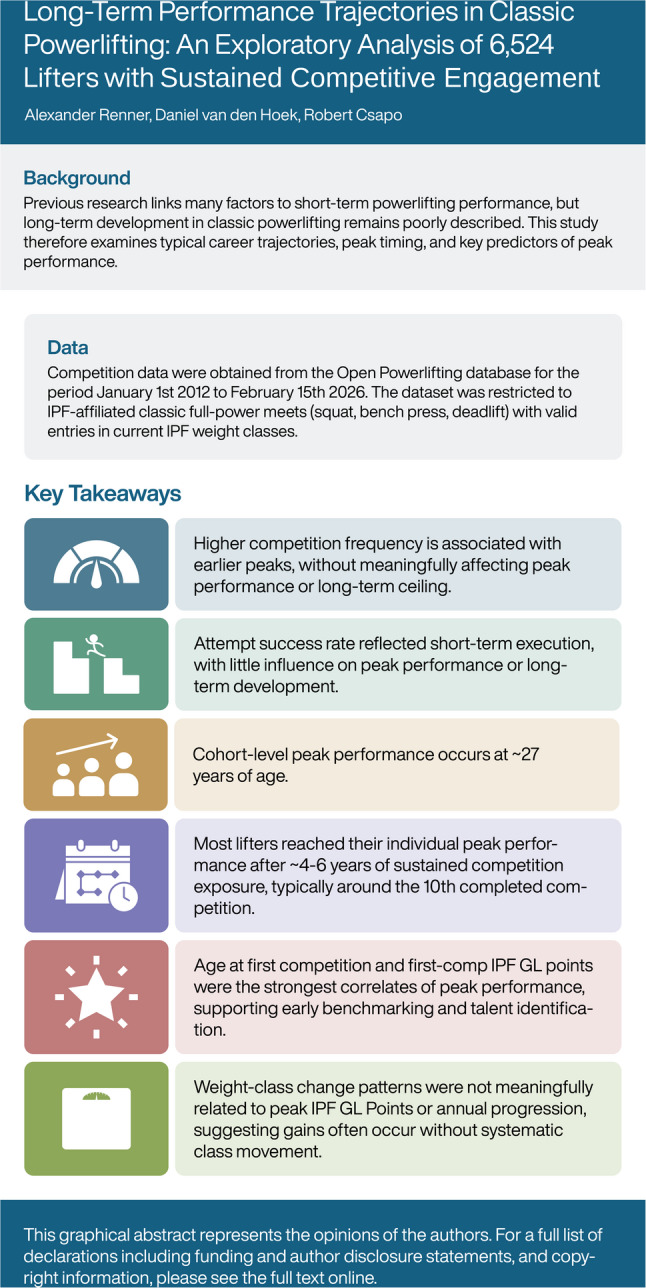

## Background

Powerlifting is a strength sport in which success is determined by the maximal loads lifted in the squat, bench press, and deadlift under standardized rules. Athletes are allowed three progressively heavier attempts per lift, and the highest successful attempt in each is summed to produce the competition total. Within each weight-class, the athlete with the highest total is the winner [1]. Because absolute competition totals are strongly influenced by body mass, performance comparisons across weight-classes require normalization [2–4]. In International Powerlifting Federation (IPF) competitions, this is achieved using a relative point system that adjusts totals for body mass and sex. IPF Good Lift (GL) Points are the official method for cross-weight-class and cross-sex performance comparisons [1].

Contemporary powerlifting comprises two competition formats: equipped and classic [5]. Equipped powerlifting permits supportive equipment such as squat and deadlift suits, bench press shirts, and knee wraps, whereas classic powerlifting allows only minimal supportive equipment [1]. Since its introduction to IPF competition in 2012, classic powerlifting has become the dominant format and provides a large, relatively homogeneous setting for examining performance development.

Although competition outcomes are defined by a single total, performance reflects multiple athlete- and context-related factors. Previous research has examined competitive powerlifting performance across domains including anthropometrics [6] and body composition [7]; competition strategies [8–12] and competition frequency [13]; age [5, 14–16]; training variables [17–19]; biomechanics [20–23]; nutrition and supplementation [23]; short term body mass manipulation [24]; injury [25–27]; coaching [28]; and psychological characteristics [23, 29, 30]. Collectively, this work has substantially advanced understanding of competitive performance correlates at the level of individual competitions or short observation periods.

Despite progress in understanding factors influencing competitive performance, long-term athletic development in classic powerlifters with sustained competitive engagement remains poorly described. This study addresses that gap by characterizing within-career performance trajectories, peak-performance timing, and factors associated with performance development using large-scale competition data. Specifically, it examines how sex, age at first competition, early-career performance, competition frequency, cumulative competition exposure, and weight-class change patterns relate to within-career trajectories and peak outcomes.

These questions are relevant for coaches and lifters. Clearer benchmarks for performance progression, peak timing, and factors associated with higher performance ceilings may support more realistic long-term planning, benchmarking, goal setting, competition scheduling, and individualized athlete development.

## Methods

This study was a retrospective observational analysis of publicly available competition data and was designed to estimate associations rather than causal effects. Competition data were obtained from the OpenPowerlifting database and restricted to IPF-affiliated competitions. The dataset was downloaded on 15 February 2026 as a comma-separated values file of individual competition entries.

### Data filtering and preprocessing

Competition data were restricted to IPF-affiliated competitions held from 2012 onward. Only classic full-power competitions (squat, bench press, and deadlift; SBD) were retained. Entries without recorded weigh-in body mass or numeric IPF GL Points were excluded. Sex was normalized, and only entries coded as female or male were retained. A completed competition was defined as an entry with a valid total, numeric IPF GL Points, and without disqualification or no-show status.

To characterize long-term trajectories, the primary analytic cohort was restricted to lifters with at least 10 completed competitions, and all eligible entries for those lifters were retained. This threshold was intended to define a cohort with sustained competitive engagement and sufficient longitudinal depth for analyses relative to observed career peak, rather than to represent the full population of IPF-affiliated classic lifters [31]. Cohort selection was quantified by comparing included and non-included otherwise eligible lifters on first-competition characteristics. In addition, a broader-sample sensitivity analysis repeated the annual progression model in all otherwise eligible lifters with at least one consecutive-year transition in annual-best IPF GL Points.

Because the 72-kg weight-class was replaced by the 69-kg and 76-kg classes on 1 January 2021, entries from the former 72-kg class were reclassified by weigh-in body mass (≤ 69.0 kg to 69 kg; >69.0 kg and ≤ 72.0 kg to 76 kg). Only current IPF weight-classes were included in the final analysis.

### Performance and career metrics

Annual best performance was defined as the maximum IPF GL Points achieved by each lifter within a calendar year. Year-to-year change in performance (Δ IPF GL Points) was calculated within lifters as the difference between consecutive annual best values to reflect comparable 12-month progression and avoid confounding by multi-year competition gaps. Years in sport were defined as years elapsed since a lifter’s first recorded competition, with the first year indexed as year 1.

Within-career trajectories were expressed relative to each athlete’s peak career performance, defined as the maximum IPF GL Points achieved across all included competitions. IPF GL Points were used as the primary performance metric because it is the official IPF relative-strength measure and permits comparison across weight-classes and sexes within the present IPF-restricted dataset. Relative performance was expressed as a percentage of peak career performance. Competition number represented the chronological sequence of competitions within each lifter’s career (1 = first recorded competition), and trajectories were examined as a function of both years in sport and competition number.

Competition frequency was defined at the lifter-year level as the number of completed competitions per calendar year (CompetitionsInYear). For athlete-level analyses, average competition frequency (AvgCompsPerYear) was calculated as the mean of CompetitionsInYear across active competition years only. For grouped comparisons, AvgCompsPerYear was rounded and categorized into seven levels (1, 2, 3, 4, 5, 6, and > 6 competitions·year⁻¹). For continuous analyses, the unrounded value was retained.

Weight-class change pattern was classified as no change, upward change, downward change, or both upward and downward change relative to the weight-class at a lifter’s first recorded competition. Strength level at first competition and peak career strength level were defined using quartiles of IPF GL Points.

Age group was categorized according to official IPF divisions: Sub-Junior (< 18 years), Junior (18–23 years), Open (24–39 years), and Masters (≥ 40 years).

Attempt success rate was derived from all recorded squat, bench press, and deadlift attempts (1–3). Non-zero attempts were treated as valid; positive values indicated success and negative values indicated failure, in accordance with OpenPowerlifting conventions. Year-level attempt success rate was calculated for each lifter-year as the proportion of successful attempts across all competitions in that calendar year, with lifter-years containing no recorded attempts treated as missing. Career attempt success rate was calculated for each lifter across the full filtered competition history. A competition-level attempt success rate was also calculated for each competition entry to analyze performance variation across competitions. Entries lacking attempt-level data and containing only best successful lifts were excluded from analyses requiring attempt success rates.

### Statistical analyses

Age-performance relationships within the primary analytic cohort were evaluated using a locally weighted smoothing curve (LOESS) applied to IPF GL Points as a function of age across all competition entries with available age data (frac = 0.15). This analysis was descriptive; uncertainty around the smoothed curve and peak age was quantified using a lifter-level cluster bootstrap to account for repeated entries within athletes. A sensitivity analysis repeated the LOESS using one annual-best observation per lifter-year.

Between-group differences in annual performance progression were examined using a multivariable ordinary least squares model with consecutive-year change in annual best IPF GL Points (Δ IPF GL Points) as the dependent variable and lifter-clustered standard errors to account for repeated transitions within athletes. Δ IPF GL Points were computed only for consecutive calendar years. Year-in-sport transition groups with fewer than 200 unique lifters were excluded from Fig. 3 and from the annual progression model. Categorical predictors were sex, age group (< 18, 18–23, 24–39, ≥ 40 years), yearly competition frequency, weight-class change pattern, and strength level at first competition. Prior-year annual-best performance (PrevBestIPFGLPoints) and year-level attempt success rate (YearSuccessRate) were included as continuous predictors. Results are reported as regression coefficients with cluster-robust standard errors and p-values and are interpreted as adjusted associations within the observational cohort. To assess robustness to the competition-threshold restriction, the same model pipeline was repeated in a broader sensitivity sample including all otherwise eligible lifters with at least one consecutive-year transition.

Within-career performance trajectories were examined using two alignments: year in sport and competition number. Performance was expressed relative to each lifter’s peak as percentage of individual peak IPF GL Points (PctOfPeakGL), calculated as 100 × (IPF GL Points / career-best IPF GL Points). For the year-in-sport alignment (Fig. 5), trajectories were based on annual best IPF GL Points within each year in sport, indexed from the calendar year of the first eligible competition (YearInSport = Year − min(Year) + 1). For the competition-number alignment (Fig. 6), trajectories were based on all entries indexed by chronological competition number within lifter (CompNumber). At each time point, distributions were summarized descriptively using the median and distribution bands (1–99%, 10–90%, and interquartile range), together with the number of contributing lifters; time points with fewer than 200 lifters were not plotted. These analyses were not repeated in the broader sample because alignment relative to observed career peak requires sufficient longitudinal exposure; in shorter careers, peak-relative trajectories would be dominated by incomplete follow-up rather than long-term development.

Peak timing was analyzed at the lifter level using one observation per athlete. Observed PeakYearInSport and observed PeakCompNumber were defined as the year in sport or competition number at which a lifter first achieved their career-best IPF GL Points; when multiple competitions shared the maximum value, the earliest occurrence was used. Associations with observed peak timing were examined using the original multivariable ordinary least squares (OLS) models with sex, age group at career entry (< 18, 18–23, 24–39, ≥ 40 years; derived from the earliest available non-missing age), average competition frequency category (1–6 and > 6 competitions per active year), weight-class change pattern, strength level at first competition (IPF GL quartiles), and career attempt success rate as predictors. To address the event-like nature of peak timing and the possibility that some observed peaks occurred at the end of follow-up, complementary discrete-time event-history models were added as robustness analyses for both alignments. For year in sport, the model used one row per lifter-year, a complementary log-log link, a flexible baseline hazard defined by year-in-sport categories, and baseline predictors only (sex, age group at career entry, and first-competition IPF GL quartile); lifters whose earliest observed peak occurred in their final observed year in sport were treated as right-censored at that terminal interval. For competition number, the corresponding model used one row per lifter-competition, a complementary log-log link, a flexible baseline hazard defined by competition-number categories, and baseline predictors only; lifters whose earliest observed peak occurred at their final observed competition were treated as right-censored at that terminal interval. Because these complementary models were specified using baseline predictors only, they were used to evaluate the robustness of the timing framework rather than to replace the original OLS analyses of full-career correlates. The OLS models therefore remained the primary analytical framework because they allowed the same full-career predictor set, including average competition frequency, weight-class change pattern, and career attempt success rate, to be examined in relation to observed peak timing. The event-history models were used specifically to assess whether the main timing interpretation was sensitive to the event-like nature of peak timing and potential right-censoring of terminal observed peaks.

Short-term within-career performance variation related to competition execution was examined using within-lifter demeaned OLS models relating deviations in attempt success rate to deviations in PctOfPeakGL. Attempt success rate was computed at the lifter-year level for year-in-sport analyses (YearSuccessRate) and at the entry level for competition-number analyses (EntrySuccessRate) using identical attempt definitions. All variables were centered within lifters so coefficients reflected deviations from each athlete’s career mean. Models were estimated with and without adjustment for career stage (YearInSport or CompNumber), with cluster-robust standard errors applied at the lifter level to account for repeated observations.

Correlates of peak career performance were assessed at the lifter level using an OLS model with peak career IPF GL Points (maximum across competitions) as the outcome. Predictors were age and IPF GL Points at first competition, sex, weight-class change pattern, career attempt success rate, and average competitions per active year. To mitigate multicollinearity among participation-exposure measures, variance inflation factors were evaluated and a parsimonious exposure metric was retained.

OLS models were estimated on complete cases. Repeated-observation OLS models used cluster-robust standard errors at the lifter level, whereas lifter-level OLS models used heteroskedasticity-consistent (HC3) standard errors. Predictor importance was quantified using Type-II ANOVA decomposition with effect sizes reported as partial η², presented as partial η² × 100 where applicable. Partial η² was interpreted as a model-specific effect-size summary rather than as an additive partitioning of explained variance. Statistical significance was set at α = 0.05. Given the large sample size, model interpretation emphasized the magnitude and practical relevance of estimated associations in addition to statistical significance.

All preprocessing and statistical analyses were conducted using custom Python scripts [32].

## Results

### Data preprocessing

**Fig. 1:**
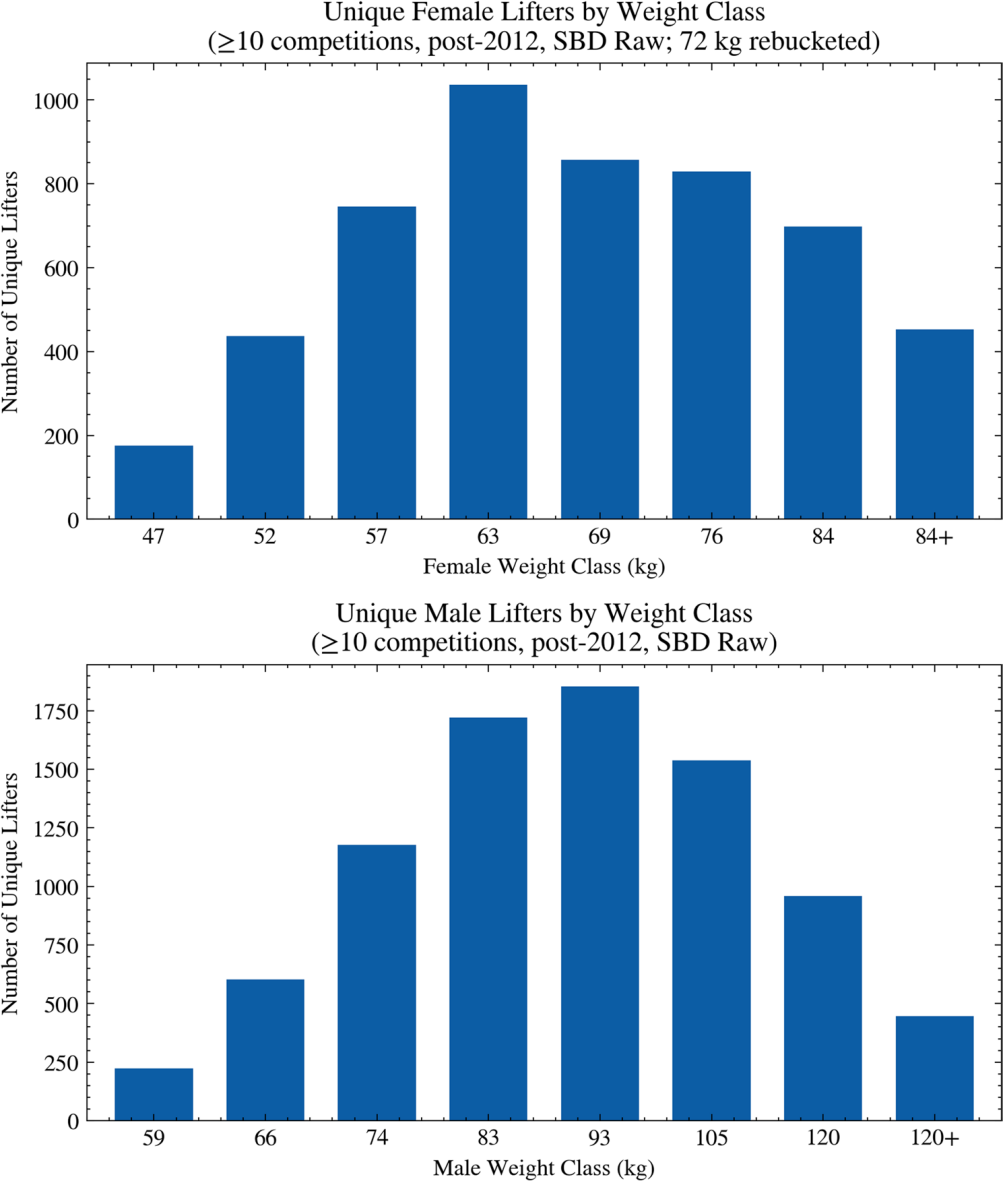
Distribution of included lifters across current IPF weight-classes, stratified by sex

**Fig. 2:**
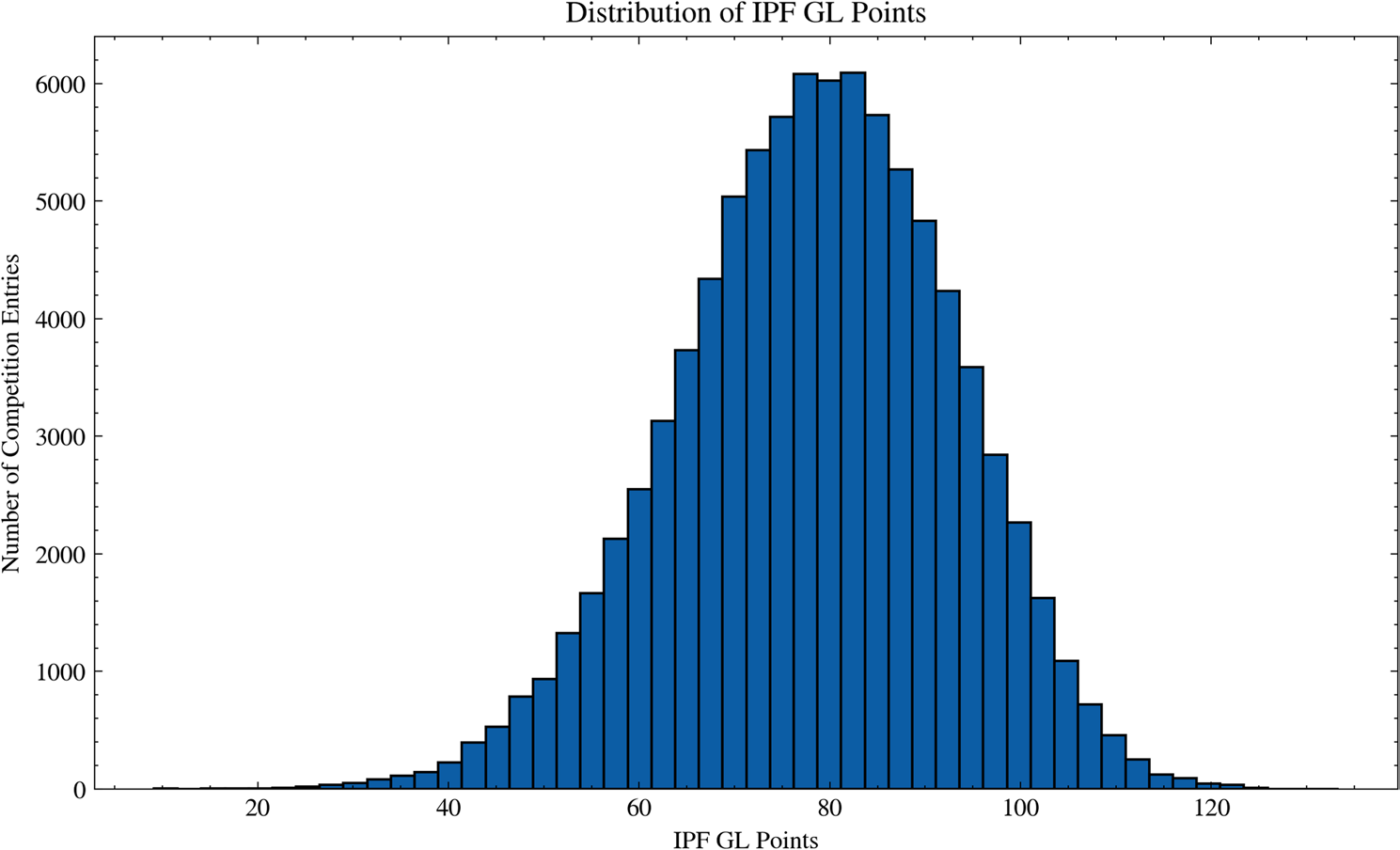
Distribution of IPF GL Points across all included competition entries

Before application of the inclusion and exclusion criteria, the downloaded dataset comprised 420,069 individual lifters and 1,438,131 competition entries. After restriction to classic SBD competitions since 2012 in the current sex-specific IPF weight-classes and exclusion of entries without numeric IPF GL Points, the base cleaned dataset comprised 201,734 lifters and 526,886 competition entries. Applying the primary ≥ 10-completed-competition threshold yielded the final analytical cohort of 6,524 lifters with sustained competitive engagement and 89,827 competition entries, of whom 2,466 were female and 4,058 were male. Compared with otherwise eligible lifters with fewer than 10 completed competitions, included lifters were older and slightly stronger at first competition and more often female (age at first competition: 30.03 ± 13.63 vs. 25.94 ± 10.23 years; first-competition IPF GL Points: 68.90 ± 12.86 vs. 65.61 ± 12.71; female: 37.8% vs. 31.0%). The distribution of lifters across weight-classes is shown in Fig. 1, and the distribution of IPF GL Points across all competition entries is shown in Fig. 2.

### Annual performance progression

**Fig. 3:**
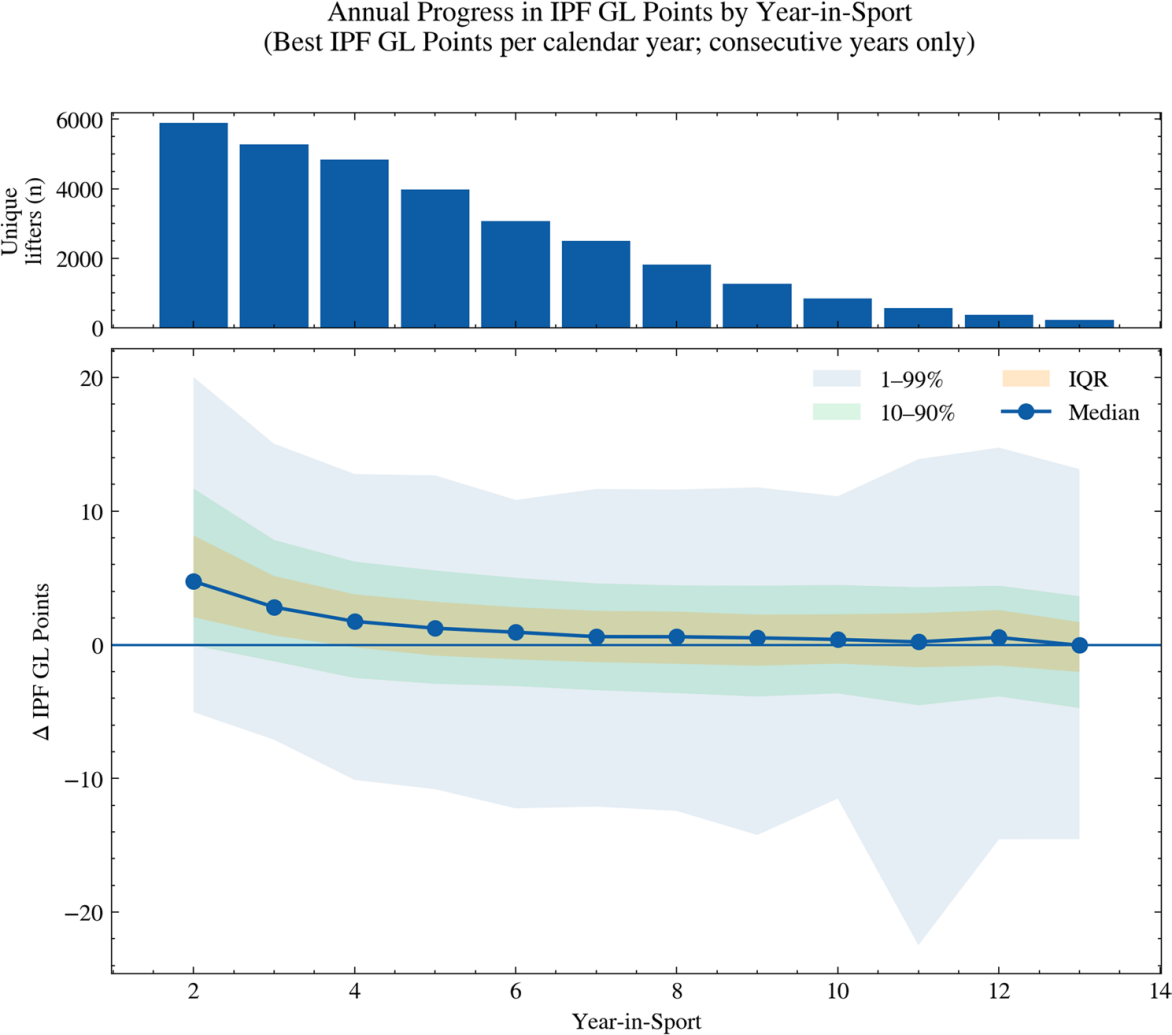
Year-on-year change in annual best IPF GL Points by year in sport. Median change and distributional ranges are shown; the upper panel indicates sample size

Year-on-year performance progression exhibited a clear downward overall trend across the athletic career, with median annual changes in IPF GL Points generally decreasing with increasing years in sport (Fig. 3).

After exclusion of year-in-sport transition groups with fewer than 200 unique lifters, 30,634 year-to-year transitions from 6,518 lifters were available for descriptive summaries; the complete-case multivariable model was fitted on 24,020 transitions from 5,726 lifters. Descriptively, median annual changes increased monotonically with higher yearly competition frequency, from 0.33 IPF GL Points for lifters completing one competition per year to 1.82 (2 competitions), 2.79 (3), 3.73 (4), 4.68 (5), 5.20 (6), and 5.44 IPF GL Points (> 6 competitions). The multivariable cluster-robust OLS model showed a significant association between annual progression and yearly competition frequency. Relative to lifters completing one competition in the year, the estimated differences in Δ IPF GL Points were + 2.02 (2 competitions), + 3.09 (3), + 3.75 (4), + 4.46 (5), + 4.75 (6), and + 5.25 (> 6), all *p* < 0.001.

Median annual changes also declined across age groups, from 6.54 IPF GL Points in lifters younger than 18 years to 3.67 in those aged 18–23 years, 1.76 (24–39 years), and 0.78 in lifters aged 40 years and above. After adjustment for the other model predictors, and using 18–23 years as the reference category, the estimated differences were + 0.88 for lifters younger than 18 years (*p* < 0.001), − 1.36 for the 24–39 year-olds (*p* < 0.001), and − 4.45 for those aged 40 years and older (*p* < 0.001).

Higher prior-year annual-best performance was associated with smaller subsequent improvements. In the adjusted model, each 1-point higher prior-year annual best IPF GL Points were associated with a 0.156 IPF GL Points smaller year-to-year change (*p* < 0.001). Year-level attempt success rate showed a positive association with annual progression (β = 5.29 per 1.0 increase in success rate; *p* < 0.001), corresponding to an expected difference of ~ 0.53 IPF GL Points in annual change per 0.10 higher success rate.

Men exhibited slightly lower annual progression than women (β = −0.85; *p* < 0.001). Early-career strength was positively associated with annual progression, with higher first-competition strength quartiles showing progressively larger annual gains relative to the lowest quartile (Q2: +1.33; Q3: +2.11; Q4: +2.92; all *p* < 0.001). In contrast, weight-class change pattern was not meaningfully associated with annual performance progression in the primary multivariable model (all *p* > 0.30). A broader-sample sensitivity analysis including all otherwise eligible lifters with at least one consecutive-year transition (93,949 complete-case transitions from 51,529 lifters) yielded materially similar association patterns. Complete sensitivity-analysis results are provided in the online repository [32].

### Timing of peak performance

**Fig. 4:**
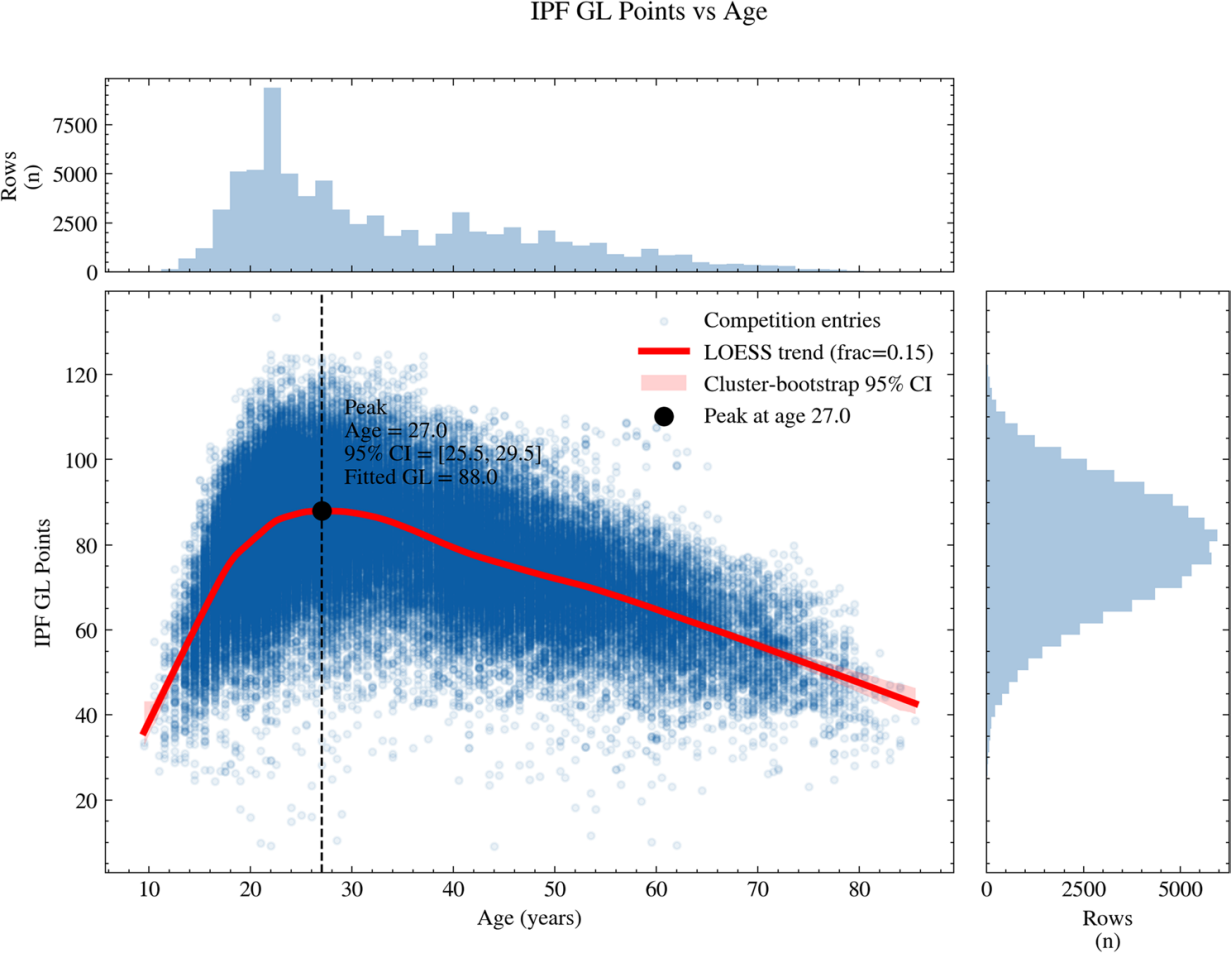
IPF GL Points as a function of age across all competition entries, with a LOESS-smoothed trend curve, lifter-cluster-bootstrap 95% confidence band, and marginal distributions

Using all competition entries with available age information, a LOESS curve with a lifter-level cluster bootstrap was applied to describe the age-performance relationship within the selected cohort. Based on 78,684 competition entries from 6,079 lifters, the smoothed curve indicated a cohort-level peak performance at 27.0 years of age (95% CI 25.5 to 29.5), with a fitted peak value of 88.0 IPF GL Points (Fig. 4). A sensitivity analysis using one annual-best observation per lifter-year yielded a similar estimate, with a peak at 28.1 years (95% CI 26.1 to 29.0) and a fitted peak value of 88.7 IPF GL Points.

**Fig. 5:**
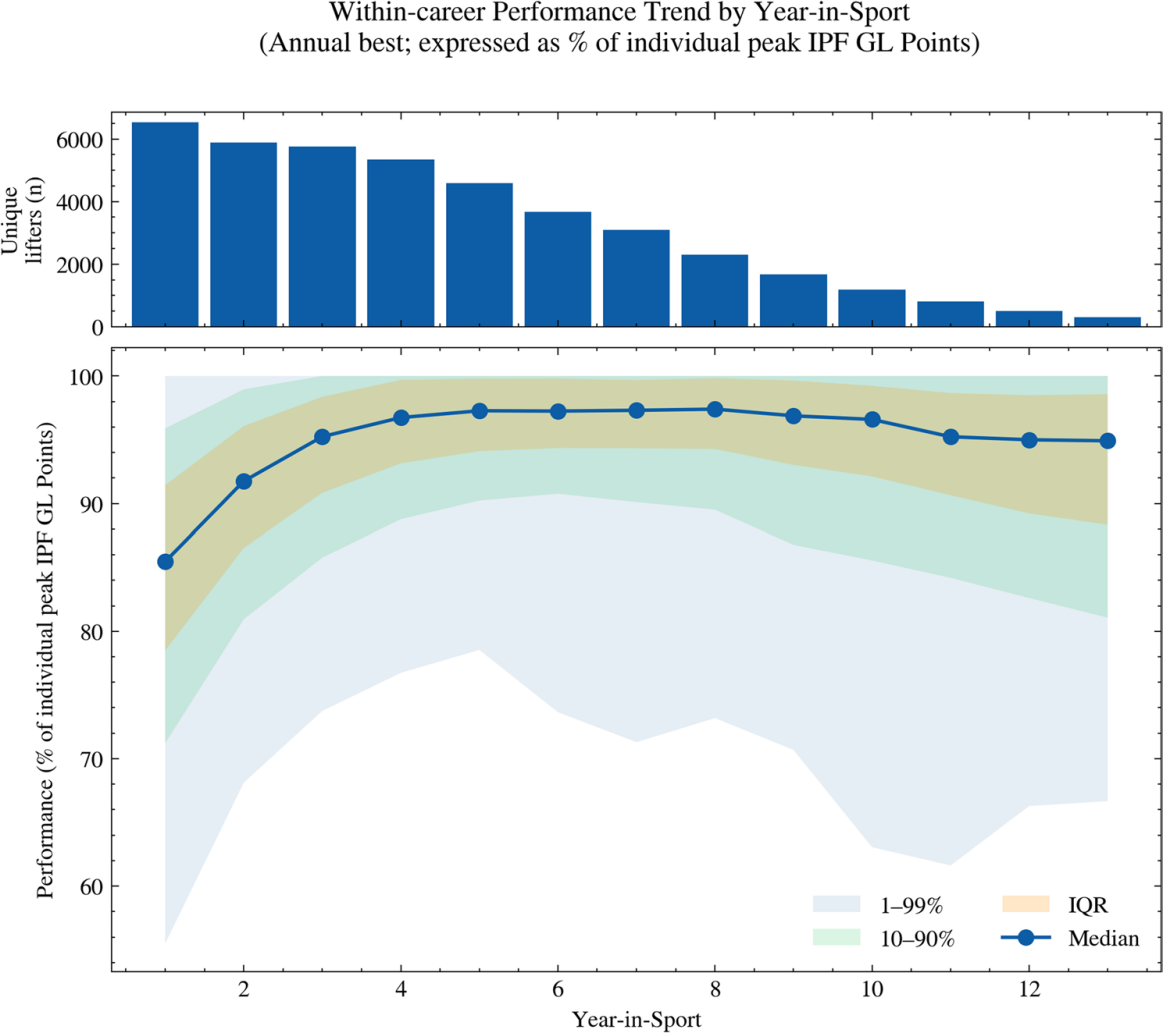
Within-career performance trajectory by year in sport, expressed as a percentage of individual peak IPF GL Points. Median and distributional ranges are shown; the upper panel indicates sample size

While the LOESS analysis describes the age-performance relationship within the selected cohort, subsequent analyses examined development relative to each lifter’s own peak. When aligned by year in sport (Fig. 5), median annual-best performance increased rapidly in the early career, remained close to individual peak levels throughout the middle years, and declined gradually in later career stages. Sample size decreased with increasing year in sport, and variability widened in the later years.

Peak timing expressed as observed PeakYearInSport was evaluated at the lifter level using one observation per athlete. In the multivariable OLS model (*N* = 5,857 complete cases), predictors jointly explained 11.1% of the variance in observed PeakYearInSport (adjusted R² = 0.108). Competition frequency accounted for the largest share of explained variance (Type II ANOVA: F = 61.51, *p* < 0.001, partial η² = 0.0594), with higher competition frequency categories associated with earlier peak timing. Age group at career entry was also independently associated with peak timing (F = 73.66, *p* < 0.001, partial η² = 0.0365), with lifters entering at age ≥ 40 years peaking earlier than those entering before age 18. Weight-class change pattern explained a modest additional share of variance (F = 15.35, *p* < 0.001, partial η² = 0.00783), whereas strength level at first competition and career attempt success rate showed only small associations. Sex was not independently associated with PeakYearInSport.

To complement this analysis, a discrete-time event-history model was fitted as a robustness analysis to account for the event-like nature of peak timing and incomplete follow-up. This model included 33,742 person-year observations from 6,079 lifters, with 3,508 observed peak events and 2,571 terminally censored lifters (42.3%). In this censoring-aware model, older age categories at career entry, the highest first-competition strength quartile, and male sex showed positive associations with earlier peak occurrence. Because this complementary model was specified using baseline predictors only, it was used to assess robustness to the event-like nature of peak timing and potential terminal right-censoring, rather than to replace the original OLS analysis of full-career correlates.

Additional within-lifter models indicated that year-to-year deviations in attempt success rate were statistically associated with deviations in performance relative to peak, but the explanatory value of this association was small. After adjustment for within-lifter deviations in year in sport, the association was attenuated but remained statistically detectable, indicating that attempt success rate primarily reflects modest short-term execution-related fluctuations within athletes rather than major differences in long-term development.

Overall, these results indicate that competition frequency is the strongest multivariable correlate of earlier observed peak timing by year in sport, while the complementary event-history analysis supports the robustness of the timing framework.

**Fig. 6:**
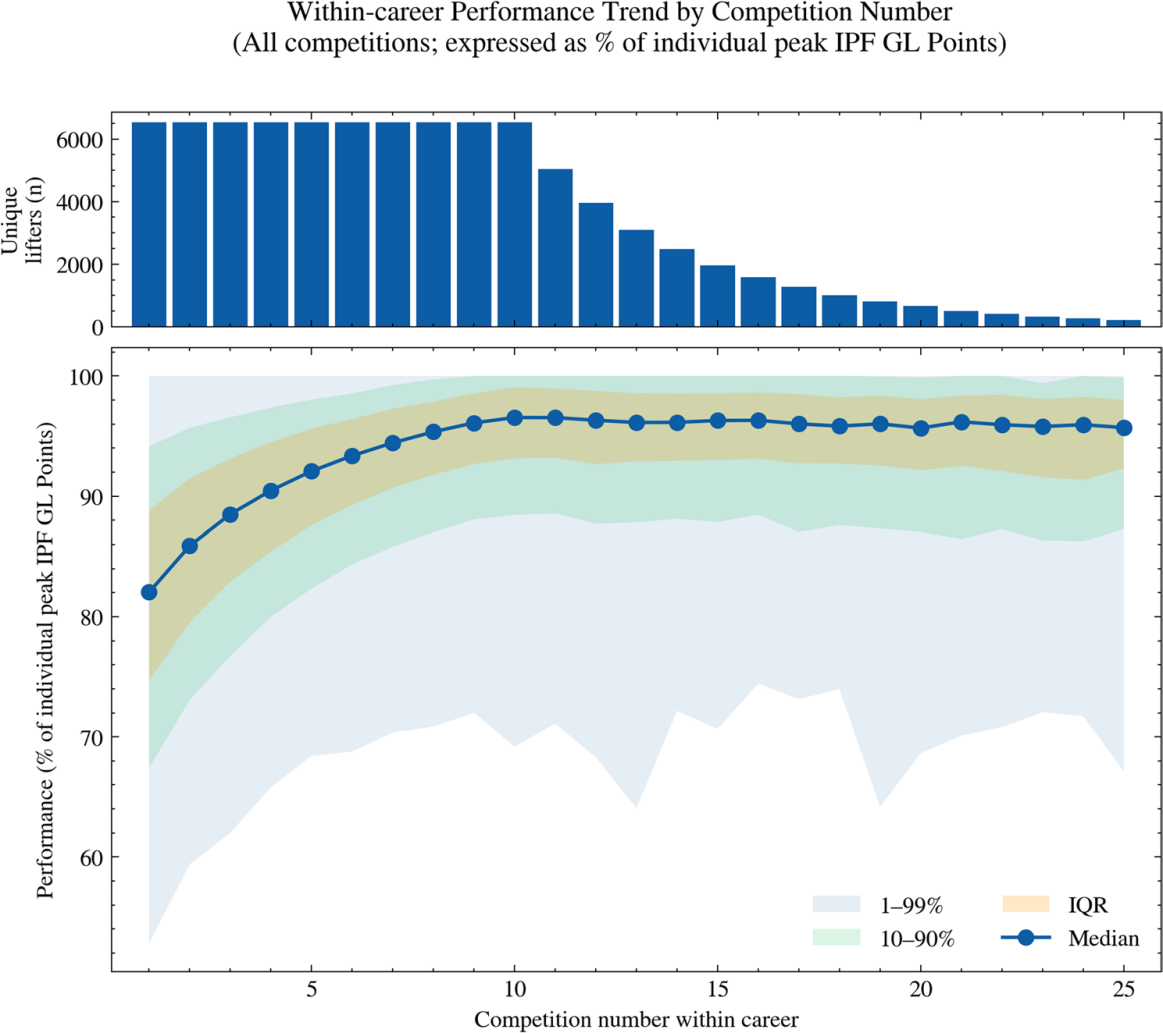
Within-career performance trajectory by competition number, expressed as a percentage of individual peak IPF GL Points. Median and distributional ranges are shown; the upper panel indicates sample size

Because years in sport may not fully reflect competitive exposure, performance was also aligned by competition number (Fig. 6). Median performance increased rapidly across the early competitions, approached individual peak levels within the first several appearances, and then remained close to peak levels thereafter, with only a slight decline in later competitions. As expected from the inclusion criterion, all 6,524 lifters contributed through competition number 10; sample size declined thereafter, and only competition numbers with at least 200 lifters are shown.

Peak timing expressed as observed PeakCompNumber was evaluated at the lifter level using one observation per athlete. In the multivariable OLS model (*N* = 5,857 complete cases), predictors jointly explained 14.7% of the variance in observed PeakCompNumber (adjusted R² = 0.144). Competition frequency accounted for the largest share of explained variance (Type II ANOVA: F = 88.64, *p* < 0.001, partial η² = 0.0835), with higher competition-frequency categories associated with later observed peak competition numbers. Age group at career entry was the next strongest correlate (F = 74.66, *p* < 0.001, partial η² = 0.0369), with older entry age associated with earlier peak timing. Weight-class change pattern, sex, and career attempt success rate showed smaller but statistically detectable associations, whereas strength level at the first competition was not independently associated with PeakCompNumber in the multivariable model.

To complement this analysis, a discrete-time event-history model was fitted as a robustness analysis to account for the event-like nature of peak timing and incomplete follow-up. This model included 65,231 person-competition observations from 6,079 lifters, with 4,499 observed peak events and 1,580 terminally censored lifters (26.0%). In this censoring-aware model, male sex and older age at career entry remained associated with earlier peak occurrence, while evidence for baseline strength was limited to the highest first-competition strength quartile. Because this complementary model was specified using baseline predictors only, it was used to assess robustness to the event-like nature of peak timing and potential terminal right-censoring, rather than to replace the original OLS analysis of full-career correlates.

Additional within-lifter models indicated that meet-to-meet deviations in attempt success rate were positively associated with deviations in performance relative to peak, but the magnitude of this association was small and attenuated after adjustment for competition number. This suggests that attempt success rate primarily reflects modest short-term execution-related fluctuations within athletes rather than major differences in long-term development.

Overall, these results indicate that, when performance is aligned by competition number, higher competition frequency is the strongest multivariable correlate of later observed peak timing, while the complementary event-history analysis supports the robustness of the timing framework.

### Peak career performance correlates

Peak career performance was quantified for each lifter as the maximum IPF GL Points achieved across all recorded competitions. The analysis was performed at the lifter level (one observation per athlete), and the final complete-case sample comprised *N* = 5,857 lifters.

**Fig. 7:**
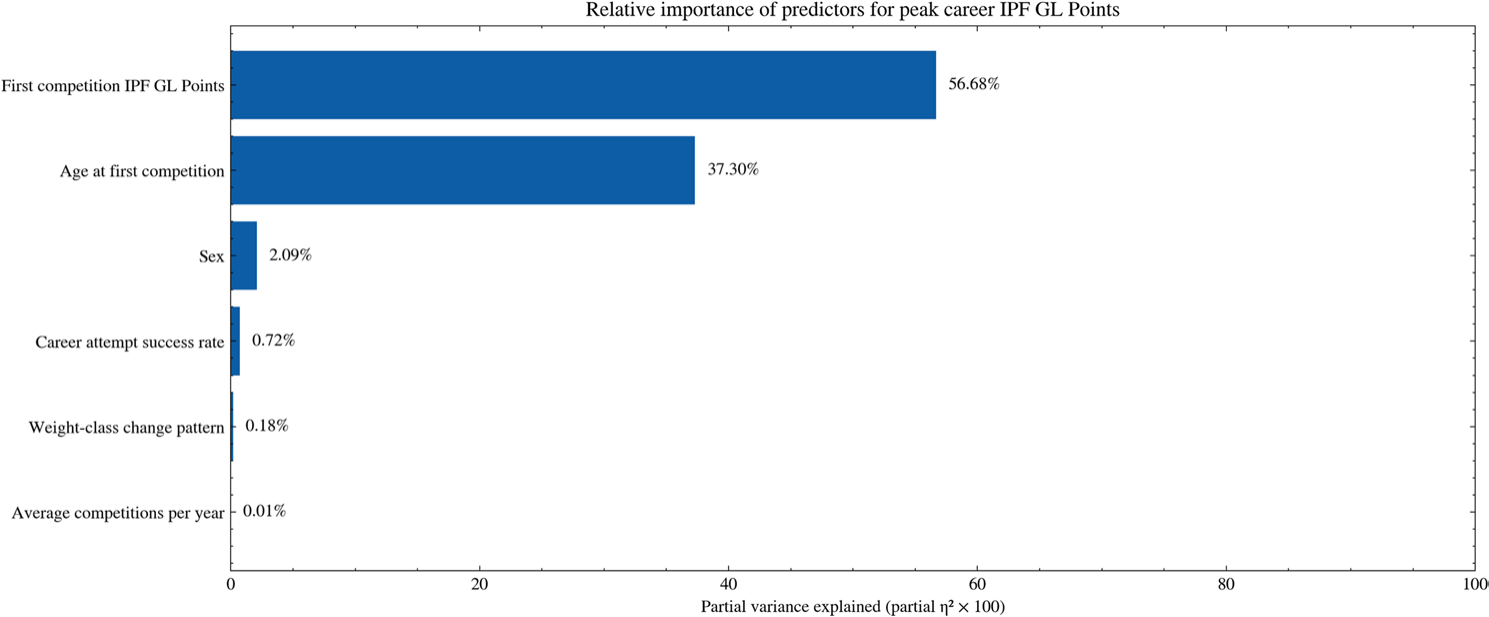
Model-specific predictor effect-size summary for peak career IPF GL Points. Bars show partial η² × 100 from a Type-II ANOVA decomposition of the fitted multivariable OLS model

A single multivariable ordinary least squares (OLS) model was fitted with peak career IPF GL Points as the outcome. Predictors were age at first competition and IPF GL Points at first competition (both continuous), sex (female, male), weight-class change pattern (no change, up, down, up & down), career attempt success rate (continuous), and average competitions per active year (continuous). To address potential multicollinearity in participation exposure, variance inflation factors (VIF) were evaluated for the included numeric predictors; average competitions per active year was retained as the exposure metric in the final specification. VIF values for the numeric predictors in the final specification were close to 1 (approximately 1.00–1.06), indicating negligible multicollinearity in the retained model. Coefficient inference was based on heteroskedasticity-consistent HC3 standard errors. Model-specific predictor effect sizes were summarized using a Type-II ANOVA decomposition of the classical OLS model, with partial η² presented as partial η² × 100 in Fig. 7.

The final model showed high overall fit (R² = 0.715; adjusted R² = 0.714). This should be interpreted primarily as indicating strong predictive concentration within the observed cohort rather than a comprehensive explanation of the processes determining peak career performance. The Type-II decomposition indicated that early competitive performance and entry age accounted for the largest partial η² effect sizes within the multivariable model. IPF GL Points at the first competition showed the largest model-specific effect size in the multivariable model (partial η² = 0.567); a 10-point higher first-competition score was associated with an estimated 7.1-point higher peak career score (β = 0.711; *p* < 0.001). Age at first competition showed the second largest model-specific effect size (partial η² = 0.373); each additional year of age at entry was associated with an estimated 0.44-point lower peak career score (β = −0.439 per year; *p* < 0.001).

Beyond first-competition performance and age at entry, other covariates contributed little additional explanatory value: sex and attempt success rate showed small associations, weight-class change effects were minor, and competition frequency showed no independent association.

## Discussion

The present investigation provides a comprehensive longitudinal analysis of performance trajectories in classic powerlifters with sustained competitive engagement, extending current understanding through a large, carefully curated IPF-restricted dataset. This design offers a detailed perspective on long-term trajectories, observed peak-performance timing, and correlates of peak career performance in this selected cohort.

### Competition frequency and career development

Previous research in powerlifting suggests that higher competition frequency is associated with superior competitive outcomes, although gains may diminish beyond approximately four competitions annually [13]. The present investigation extends this work by distinguishing between the role of competition frequency in observed peak timing and its association with peak-performance magnitude.

Competition frequency showed an alignment-dependent association with observed peak timing. When timing was indexed by year in sport, higher competition frequency categories were associated with earlier observed PeakYearInSport. When timing was indexed by competition number, higher competition frequency categories were associated with later observed PeakCompNumber. Taken together, this pattern suggests that lifters who competed more frequently tended to accumulate more competition exposures before reaching their observed peak, but did so over a shorter elapsed time. This pattern is broadly consistent with observations in other strength sports, including Olympic weightlifting, where greater competition exposure and earlier specialization have been associated with earlier peak ages and more rapid early development [15]. Similar principles have been described more broadly in strength–power sports, where competition provides a context for skill refinement, psychological adaptation, and calibration of training strategies [33], although these mechanisms were not directly tested here. The complementary event-history analyses were consistent with the main timing framework but did not remove the observational nature of these associations.

In contrast, when peak career IPF GL Points were examined in a multivariable framework, average competitions per active year showed no independent association with peak-performance magnitude once age at entry and IPF GL Points at first competition were accounted for. This suggests that, within the observed cohort, competition frequency was associated more with the pacing and competitive exposure of development than with peak-performance magnitude. Peak magnitude may instead reflect a broader set of factors not captured in the present dataset, including prior training history, training quality, accumulated adaptation, biological constraints, injury history, and selection into longer competitive careers [33, 34]. Accordingly, the present results should not be interpreted as evidence that higher competition exposure increases peak career performance.

Taken together, these findings suggest that competition frequency may be more informative as a marker of developmental pacing and competitive exposure than as an independent driver of peak performance. This interpretation may inform flexible competition scheduling in sustained competitors, while remaining consistent with strength-sport evidence and long-term athlete development models emphasizing strategic rather than maximal competition exposure [35, 36].

### Sex differences and interpretation of IPF GL points

The present investigation found that female lifters achieved slightly higher peak IPF GL Points than male lifters in the multivariable model. This is unlikely to reflect superior relative strength among women and more plausibly reflects the sex-specific normalization embedded in the IPF GL Points system [37]. Both in the present dataset and in previous investigations, male lifters tended to achieve higher absolute and relative strength levels than female lifters [14]. Accordingly, sex-related differences in IPF GL Points should be interpreted cautiously.

### Strength level, sex, and annual performance progression

Previous research suggests that, when training is matched, strength adaptations are broadly similar between sexes, with only small context-dependent differences [38]. In strength sport, slower improvement among stronger and more advanced lifters may parsimoniously explain apparent between-sex differences in progression [39, 40]. In the present investigation, women showed slightly greater median annual improvements, but this likely reflects baseline performance distributions, competitive maturity, and regression to the mean rather than sex-specific physiology, particularly given the trivial effect size.

Beyond between-group differences, the present analyses indicate that annual performance progression follows a non-linear trajectory across the competitive career. Declining median year-on-year changes in IPF GL Points with increasing years in sport (Fig. 3), together with within-career trajectories relative to individual peak performance (Figs. 5 and 6), show that improvements are largest early in a lifter’s career and progressively diminish with increasing competition exposure. This pattern is consistent with the possibility that early gains reflect neural [41], technical, and coordination-related adaptations [42], followed by slower improvements as athletes approach biological constraints [43], although the present observational data do not permit direct mechanistic inference. Importantly, this deceleration does not indicate an early performance ceiling, as smaller improvements remained evident in later career stages. The wide distributional ranges in Fig. 3 also indicate substantial inter-individual variability, suggesting that meaningful year-on-year improvements can still occur at more advanced stages. This pattern aligns with previous findings of rapid early gains followed by slower but continued progress [5].

In the multivariable cluster-robust OLS model of year-to-year change in annual best IPF GL Points, the strongest associations were observed for competition frequency, prior-year performance, and year-level attempt success rate. Higher yearly competition frequency showed a graded positive association with annual progression, whereas higher prior-year annual-best performance was strongly negatively associated with subsequent change, consistent with diminishing returns at higher standards. Year-level attempt success rate was also positively associated with annual progression, although its practical effect was modest.

By comparison, sex, weight-class change pattern, and strength level at first competition contributed little relative to competition frequency, prior-year performance, and execution-related indicators.

Collectively, these results indicate that, although several factors systematically influence annual performance changes, most variance remains unexplained, underscoring the multifactorial and highly individual nature of strength development. Given the large sample size, several effects reached statistical significance despite limited practical impact.

### Attempt success rate and competitive performance quality

While previous investigations suggested that successful squats and bench presses were positive predictors of relative strength, whereas successful deadlifts were not associated with greater competitive success [8], the present analyses provide a broader perspective by examining aggregated attempt success across lifts and competitive contexts. Overall success rate is a meaningful construct because it captures global meet execution, integrating technical consistency, attempt selection, and decision-making. In the present study, attempt success rate was a statistically reliable but practically minor correlate of performance development. At the year level, higher attempt success rate was associated with slightly greater annual improvements in IPF GL Points, but this effect was small relative to competition frequency and prior-year performance. Career attempt success rate likewise showed statistically detectable but practically trivial associations with peak timing and peak career performance, indicating that long-term execution quality contributes little to explaining when lifters peak or how high that peak is.

In contrast, within-lifter analyses showed that both year-to-year and competition-to-competition deviations in attempt success rate were positively associated with deviations in performance relative to individual peak, although these associations attenuated after adjustment for career stage. This suggests that attempt success rate primarily reflects short-term performance quality rather than long-term athletic development.

### Age and peak performance timing

Solberg et al. reported a peak age of 35 ± 7 years, whereas Hernández Ugalde reported 27.67–31.50 years [15, 44]. In the present analysis, peak performance occurred at 27.0 years (95% CI 25.5 to 29.5).

Together, these findings suggest that estimated peak age is sensitive to competition format and sampling frame. The older peak reported by Solberg et al. may reflect the greater prominence of equipped lifting and the selective nature of World Championship cohorts, whereas Hernández Ugalde’s younger estimate aligns more closely with the present classic-focused population-level analysis. Overall, the present results support a cohort-level peak in the late 20s in classic powerlifting, consistent with broader evidence that younger athletes typically show greater annual strength improvements than older athletes [5].

### Weight-class changes and body composition considerations

Previous studies highlighted the importance of lean body mass for powerlifting performance [7, 45–47]. The present investigation adds cohort-level context by showing that weight-class change patterns were not meaningfully associated with peak career IPF GL Points and contributed little to annual performance progression among sustained competitors, although moving up or both up and down was modestly associated with later peak timing. These findings suggest that improvements in relative performance often occur without systematic upward movement across weight-classes and may instead reflect training adaptation and body recomposition within a class rather than class changes per se, although body composition was not measured directly and causal inference is not warranted. Weight-class selection remains a multifactorial process balancing health, body composition, performance potential, preferences, short-term competitiveness, and long-term development [24].

### Methodological context and data limitations

Several previous investigations have used the OpenPowerlifting database to examine performance and participation [5, 31, 40, 48–50]. While this approach enables large-scale analyses, it also has limitations. OpenPowerlifting aggregates data from multiple federations, many without formal anti-doping regulations, raising concerns about the influence of performance-enhancing drug use on observed strength levels and progression patterns. In contrast, the IPF is a signatory to the World Anti-Doping Code and applies anti-doping rules aligned with World Anti-Doping Agency standards [51]. Restricting the dataset to IPF-affiliated competitions helps mitigate, though not eliminate, confounding from heterogeneous doping controls across federations. The additional restriction to lifters with at least 10 completed competitions provided sufficient longitudinal depth for peak-relative and peak-timing analyses, but also yielded a selected cohort of sustained competitors rather than a representative sample of all otherwise eligible lifters. While the annual progression findings were materially unchanged in a broader-sample sensitivity analysis, the trajectory and peak-timing results should be interpreted primarily as describing lifters with prolonged competitive engagement. The comparatively high R² of the peak-career-performance model should also be interpreted cautiously. It likely reflects strong predictive anchoring by first-competition IPF GL Points and age at entry, both of which already summarize substantial differences present at the beginning of observed follow-up, rather than a comprehensive explanation of the processes that determine ultimate peak performance. First-competition IPF GL Points should therefore not be interpreted as a pure marker of latent talent or long-term performance potential, as it may also reflect unmeasured prior training history, preparation quality, adaptation status, coaching, injury history, and selection into longer competitive careers. More broadly, because the study was retrospective and observational, the reported relationships, particularly those involving competition frequency and first-competition performance, should be interpreted as associations rather than causal effects and may reflect unmeasured confounding, including training quality, coaching, injury status, motivation, recovery capacity, and selection into longer competitive careers. Effective sample sizes also varied across analyses because of missing age data, absent attempt-level records, and complete-case exclusion of missing covariates.

### Temporal validity and future directions

A final limitation of the present investigation is its retrospective, observational, and time-bound nature. Strength norms and competitive standards in powerlifting evolve over time [50], so the generalizability of the present findings should be periodically re-evaluated as new competition data become available. Future research could extend this framework by incorporating additional athlete-level characteristics, including longitudinal body composition changes, more detailed training variables, injury history, and richer information on health and recovery status. Although such data are not routinely available in open competition databases, their integration is becoming increasingly feasible through emerging athlete monitoring practices, structured surveys, and federated data collection. Linking competition trajectories to these variables may help clarify underlying mechanisms, reduce residual confounding, and determine whether the present patterns remain stable as competitive standards evolve.

## Conclusion

The present investigation provides a large-scale analysis of long-term athletic development in classic powerlifting using competition histories from 6,524 lifters with sustained competitive engagement. By examining annual performance progression, observed peak timing, and correlates of peak career IPF GL Points, it provides cohort-level benchmarks and clarifies how early-career characteristics and participation patterns were associated with long-term outcomes. Performance development followed a non-linear trajectory, with larger gains early in the career and progressively smaller improvements thereafter, although meaningful progress could still occur later. Attempt success rate primarily reflected short-term competitive execution rather than long-term development. Competition frequency was associated with faster progression and with alignment-dependent observed peak-timing patterns, earlier when indexed by year in sport but later when indexed by competition number, but not with higher peak career IPF GL Points once age at entry and first-competition performance were accounted for, suggesting that competition use is more relevant to the pacing and competitive exposure of development than to peak magnitude. Many lifters approached peak performance within the first ~ 10 competitions, which may help inform early benchmarking and competition planning. Age at first competition and IPF GL Points at first competition were the strongest statistical correlates of observed peak career performance, but these associations should be interpreted as reflecting observed early-career differences rather than latent performance potential. Sex- and weight-class-related effects were small and of limited practical relevance. Overall, the observed patterns are consistent with long-term success in classic powerlifting being linked to sustained development and long-term planning, but the present observational design does not support causal conclusions about the effects of competition exposure itself.

## Data Availability

The datasets analyzed during the current study were derived from the publicly available OpenPowerlifting database. The processed dataset and analysis scripts supporting the conclusions of this article are available in the Zenodo repository at https://doi.org/10.5281/zenodo.20282050. The original raw competition data are available from OpenPowerlifting at https://www.openpowerlifting.org/ under their respective data usage terms.

## Abbreviations

ANOVA: Analysis of Variance
AvgCompsPerYear: Average competitions per active year
CompNumber: Competition number within lifter
CompetitionsInYear: Competitions completed per calendar year
CI: Confidence interval
EntrySuccessRate: Competition–entry–level attempt success rate
GL: Good Lift (IPF GL Points)
HC3: Heteroskedasticity–consistent standard errors (HC3 estimator)
IPF: International Powerlifting Federation
LOESS: Locally Estimated Scatterplot Smoothing
OLS: Ordinary Least Squares
PctOfPeakGL: Percentage of individual peak IPF GL Points
PeakCompNumber: Observed competition number at peak (earliest tie)
PeakYearInSport: Observed year–in–sport at peak (earliest tie)
PrevBestIPFGLPoints: Prior–year annual–best IPF GL Points
R²: Coefficient of determination
SBD: Squat, Bench press, Deadlift
SD: Standard deviation
VIF: Variance Inflation Factor
YearSuccessRate: Year–level attempt success rate
α: Significance level
β: Regression coefficient
Δ: Change (difference)
η²: Eta squared (effect size); partial η² reported
